# *N*^6^-Methyladenosine Methylomic Landscape of Ureteral Deficiency in Reflux Uropathy and Obstructive Uropathy

**DOI:** 10.3389/fmed.2022.924579

**Published:** 2022-06-20

**Authors:** Hua Shi, Tianchao Xiang, Jiayan Feng, Xue Yang, Yaqi Li, Ye Fang, Linan Xu, Qi Qi, Jian Shen, Liangfeng Tang, Qian Shen, Xiang Wang, Hong Xu, Jia Rao

**Affiliations:** ^1^Department of Nephrology, Children's Hospital of Fudan University, Shanghai, China; ^2^Shanghai Kidney Development and Pediatric Kidney Disease Research Center, Shanghai, China; ^3^Shanghai Key Lab of Birth Defect, Children's Hospital of Fudan University, Shanghai, China; ^4^Department of Pathology, Children's Hospital of Fudan University, Shanghai, China; ^5^Department of Urology, Children's Hospital of Fudan University, Shanghai, China; ^6^State Key Laboratory of Medical Neurobiology, Institutes of Brain Science and School of Basic Medical Science, Fudan University, Shanghai, China

**Keywords:** uropathy, megaureter, vesicoureteral reflux, m^6^A, *N*^6^-methyladenosine, microarray

## Abstract

**Background:**

Congenital anomalies of the kidneys and urinary tracts (CAKUT) represent the most prevalent cause for renal failure in children. The RNA epigenetic modification *N*^6^-methyladenosine (m^6^A) methylation modulates gene expression and function post-transcriptionally, which has recently been revealed to be critical in organ development. However, it is uncertain whether m^6^A methylation plays a role in the pathogenesis of CAKUT. Thus, we aimed to explore the pattern of m^6^A methylation in CAKUT.

**Methods:**

Using m^6^A-mRNA epitranscriptomic microarray, we investigated the m^6^A methylomic landscape in the ureter tissue of children with obstructive megaureter (M group) and primary vesicoureteral reflux (V group).

**Results:**

A total of 228 mRNAs engaged in multiple function-relevant signaling pathways were substantially differential methylated between the “V” and “M” groups. Additionally, 215 RNA-binding proteins that recognize differentially methylated regions were predicted based on public databases. The M group showed significantly higher mRNA levels of m^6^A readers/writers (YTHDF1, YTHDF2, YTHDC1, YTHDC2 and WTAP) and significantly lower mRNA levels of m^6^A eraser (FTO) according to real-time PCR. To further investigate the differentially methylated genes, m^6^A methylome and transcriptome data were integrated to identified 298 hypermethylated mRNAs with differential expressions (265 upregulation and 33 downregulation) and 489 hypomethylated mRNAs with differential expressions (431 upregulation and 58 downregulation) in the M/V comparison.

**Conclusion:**

The current results highlight the pathogenesis of m^6^A methylation in obstructive and reflux uropathy.

## Introduction

*N*^6^-methyladenosine (m^6^A) methylation is the most abundant and conserved internal modification in messenger RNA (mRNA) and long non-coding RNAs (1). m^6^A can be produced by the “writer” complex methyltransferase-like 3 (METTL3), methyltransferase-like 14 (METTL14) (2), and Wilms tumor 1-associated protein (WTAP) (3), removed by the demethylases (“erasers”) fat mass and obesity associated protein (FTO) (4) and alkB homolog 5 (ALKBH5) (5), and recognized by “readers” such as the YTH family of proteins (6). m^6^A is involved in mRNA splicing, polyadenylation, export, translation, and stability (7). Throughout life, m^6^A-modified mRNAs play a role in variety of physiological activities, developmental processes and disease pathologies (7, 8). Multiple diseases, such as heart failure (9), type 2 diabetes (10), kidney injury (11) and asthma (12) and various malignancies are associated with changes in m^6^A methylation.

Thousands of m^6^A peaks in mammalian mRNA have been identified in techniques of the high-throughput sequencing. According to the Nephroseq database (www.nephroseq.org, University of Michigan, Ann Arbor, MI) (13), hypoexpression of METTL3, METTL14 and WTAP has been reported in diabetic mice, in patients with chronic kidney disease (CKD) and patients with focal segmental glomerulosclerosis, indicating the feedback disruption of m^6^A. It has been demonstrated that METTL14-induced m^6^A methylation could posttranscriptionally modulate Sirt1 mRNA, contributing to podocyte injury(14). Other studies discovered the METTL14-YAP1 pathway participated in the renal ischemia-reperfusion injury (15) and METTL14-regulated PI3K/Akt signaling pathway is involved in renal tubular cell epithelial-mesenchymal transition in diabetic nephropathy (16). Despite recent advances in m^6^A and numerous physiological and pathological processes associated with kidney disease (10), little is known about m^6^A-mediated regulatory effect in kidney development.

Congenital anomalies of the kidneys and urinary tracts (CAKUT) are embryonic disorder that causes a spectrum of defects in the kidneys, the ureters, the bladder and the urethra during development. CAKUT affects ~5 per 1,000 live newborns and accounts for 30% to 50% of all children end-stage renal disease (ESRD) (17). CAKUT induces renal deficiency that is constantly associated with urinary tract infection (UTI) and urine outflow abnormalities. Outflow abnormalities include obstructive nephropathies caused by obstruction of ureteropelvic junction/ureterovesical junction, megaureter, posterior urethral valves, and reflux uropathy induced by vesicoureteral reflux (VUR). During nephrogenesis, genetic and epigenetic changes have been demonstrated to be important (17, 18). Even though there have been significant advances in kidney diseases, the etiology and morphogenesis of uropathy are still unknown. Megaureter might lead to functional obstruction with an adynamic ureteral ending segment. In neonates, primary obstructive megaureter is involved in the fetal ureteric folds that remain or delay in the process of peristalsis. Primary VUR is distinct from megaureter, which is caused by a short or missing intravesical ureter or other vesico-ureteric junction disruption, accompanied by normal structural and functional ureters. Early diagnosis of reflux or obstructive uropathy is critical to prevent renal damage from reflux, obstruction and infection. Due to the lack of adequate knowledge of the mechanism, it is difficult to predict the renal prognosis of children with reflux or obstructive nephropathy.

To gain a better understanding of the role of m^6^A in uropathy, we enrolled the ureter samples from patients with VUR or megaureter to investigate of the distribution schema and readout of the differentially methylated mRNA. The differential methylation site was employed to make a prediction on the RNA-binding protein (RBP) candidates. A final integrated analysis of the m^6^A microarray data was performed to determine the association between the mRNAs methylation and the gene expression levels. In this paper, we illustrated the critical role of m^6^A methylation in ureteral defects of CAKUT.

## Materials and Methods

### Patients and Human Materials

After receiving approval from the Research Ethics Board of Children's Hospital of Fudan university (2020-363), our local ethics board, patients with obstructive uropathy or reflux uropathy were enrolled into the study from January 2020 to October 2020. Prior to enrolling patients in this study, we received written informed permission from all parents or legal guardians. The diagnosis of different types of CAKUT was established through a series of radiological examinations. Primary obstructive megaureter was diagnosed by combining the clinical features and radiological findings of diuretic renography. The surgery criteria for primary obstructive megaureter were the differential renal function <40% and/or anterior–posterior diameter of the renal pelvis on ultrasonographic scan >100 mm and half-time of the elimination phase (T1/2) of 99mTc –DTPA diuretic renogram >20 min. VUR diagnosis criteria were based on the parameters that was established by the International Reflux Study Committee in 1981 (19). Exclusion criteria were associated anomalies including pelviureteric junction obstruction, ectopic ureter, duplicated collecting systems, ureterocele, posterior urethral valves, neurogenic bladder or prune belly. The children were categorized into two study groups. Patients with primary non-refluxing megaureter were distributed into the patient group of “M” (M group). Patients with primary VUR who had no other urinary tract defects were distributed into the patient group of “V” (V group). The reflux degree of these patients was greater than grade three.

Specimens were obtained from children who underwent ureteral reimplantation surgeries at our hospital. Due to the nature of subject, it was impossible to obtain tissue samples from completely normal distal ureteric ends. Therefore, there were no normal controls. We compared the pathological features among the ureter tissue samples from patients with megaureter or VUR. The surgical cut in the ureter tissue was 3–5 mm long, and roughly 1 mm tissue was removed from the distal ureter, and sent to the department of pathology. The surgeon and the pathologist made sure the complete of samples and prevented contamination. The pathologist dehydrated the tissue before embedding it in paraffin. The remaining tissues were cut into 1 mm blocks and deposited in RNA preservation solution, which was preserved at 4°C overnight before being transferred to an −80°C freezer for long-term storage.

### m^6^A mRNA Epitranscriptomic Microarray

Total RNA was isolated from each sample using TRIzol reagent (Invitrogen, Carlsbad, CA, USA) as directed by the manufacturer. The NanoDrop ND-1000 was used to determine purity and amount of total RNA samples (Thermo Fisher, Shanghai, China). To detect and verify the integrity of total RNA (RIN values >7.0), we employed a Bioanalyzer 2100 (Agilent, Santa Clara, CA, USA) for agarose electrophoresis.

Due to the RNA sample requirements for microarray and limited tissue samples from children's ureters, we utilized the m^6^A microarray (total RNA <1 ug) rather than MeRIP Seq (total RNA >120 ug). The Arraystar Human m^6^A-mRNA&lncRNA Epitranscriptomic microarray analysis was conducted in the three samples from patients with primary non-refluxing megaureter compared with the three samples from patients with primary VUR. In brief, m^6^A antibody was used to immunoprecipitate total RNAs isolated from specimens. As the “IP”, the modified RNAs were eluted from the immunoprecipitated magnetic beads. The unmodified RNAs were recovered as “Sup” from the supernatant. The Arraystar Super RNA Labeling Kit was used to label the “IP” and “Sup” RNAs with Cy5 and Cy3 respectively as cRNAs in separate procedures. Arraystar Human mRNA&lncRNA Epitranscriptomic Microarray (8x60K, Arraystar) with 44,122 mRNA degenerate probes degenerate probes was used to hybridize the cRNAs. After washing the slides, an Agilent Scanner G2505C were used to scan the arrays in two-color channels.

### Microarray Data Analysis

To analyze acquired array images, Agilent Feature Extraction software (version 11.0.1.1) was unutilized. The average value of log2-scaled Spike-in RNA intensities was presented to standardize the raw intensities of IP (immunoprecipitated, Cy5-labeled) and Sup (supernatant, Cy3-labeled). Following Spike-in normalization, the probe signals with Present (P) or Marginal (M) QC flags in the Excel sheet were kept as “All Targets Value” for further evaluation of “m^6^A methylation level,” “m^6^A amount,” and “expression level”. Based on the IP (Cy5-labeled) and Sup (Cy3-labeled) normalized intensities, the “m^6^A methylation level” was assessed for the percentage of alteration. The calculating formula was provided in the Supplementary Tables S2, S3). Based on the IP (Cy5-labeled) normalized intensities, the “m^6^A quantity” was derived for the m^6^A methylation amount. The sum of IP (Cy5-labeled) and Sup (Cy3-labeled) normalized intensities of RNA was used to calculate “expression level.” Filtering with the fold change and statistical significance (p-value) thresholds were performed to identify the differently m^6^A-methylated RNAs or differentially expressed RNAs between the two comparisons. To illustrate the distinct m^6^A-methylation or expression pattern among samples, hierarchical clustering was conducted.

### Methylated RNA Immunoprecipitation (MeRIP)-qPCR Validation

MeRIP assay was conducted for the same six RNA samples using the Magna MeRIP™ m^6^A Kit (Millipore, Billerica, MA, USA). Magnetic IP with a monoclonal antibody against m^6^A and IgG antibody was used following total RNA fragmentation into 100 nucleotides. qRT-PCR normalized the input RNA to analyze the immunoprecipitated RNA. Each experiment was performed in triplicate independently. The primers sequences with reference to the m^6^A motif regions of the mRNAs were displayed in Supplementary Table S1.

### Real-Time PCR

The m^6^A writers (METTL3, METTL14, and WTAP), readers (YTHDC1, YTHDC2, YTHDF1, YTHDF1, YTHDF2 and YTHDF3) and erasers (FTO and ALKBH5) were selected and analyzed by real-time PCR in ureteral samples. Due to the quantity restriction of tissue samples, the independent set of ureteral samples was performed the qRT-PCR. With the High Capacity RNA to cDNA Kit, cDNA was reverse transcribed from 1 μg of total RNA (Applied Biosystems). The mRNA levels were assessed by real-time PCR analysis with SYBR-green protocol by gene-specific primers (Supplementary Table S1). 18S rRNA was selected as house-keeping control. For MeRIP-qPCR analysis of differentially methylated RNAs, equal volume of immunoprecipitated RNA or 10% of input RNA was converted to cDNA and amplified using gene-specific primers for mRNAs (Supplementary Table S1). Triplicates of real-time PCR experiments were performed.

### Data Processing and Statistical Analysis

Differentially m^6^A-methylated RNAs and differentially expressed RNAs in the M/V group comparison were presented by filtering with the fold change (FC ≥ 1.5 or ≤ 0.7) and were identified at a false discovery rate (FDR) of <0.05. We retrieved a gene list from the Enrichr platform by searching for “uropathy” in the metadata including libraries created from TRRUST, BioPlanet, GWAS Catalog, the UK Biobank, ClinVar, PheWeb, and DepMap (20). The matching genes were highlighted into the m^6^A quantity and m^6^A expressing level profiling data (Supplementary Table S2). For differentially m^6^A-methylated or expressed RNAs, a hierarchical clustering heatmap was displayed. Differentially m^6^A-methylated or expressed mRNAs were analyzed for Gene Ontology (GO) analysis using the top GO package (R environment) and Kyoto Encyclopedia of Genes and Genomes (KEGG) pathway enrichment. Fisher's exact test were used to determine the differences between the two groups with a *P* < 0.05 defined as statistically significance.

## Results

### Patient Sample Characteristics

**Figure 1:**
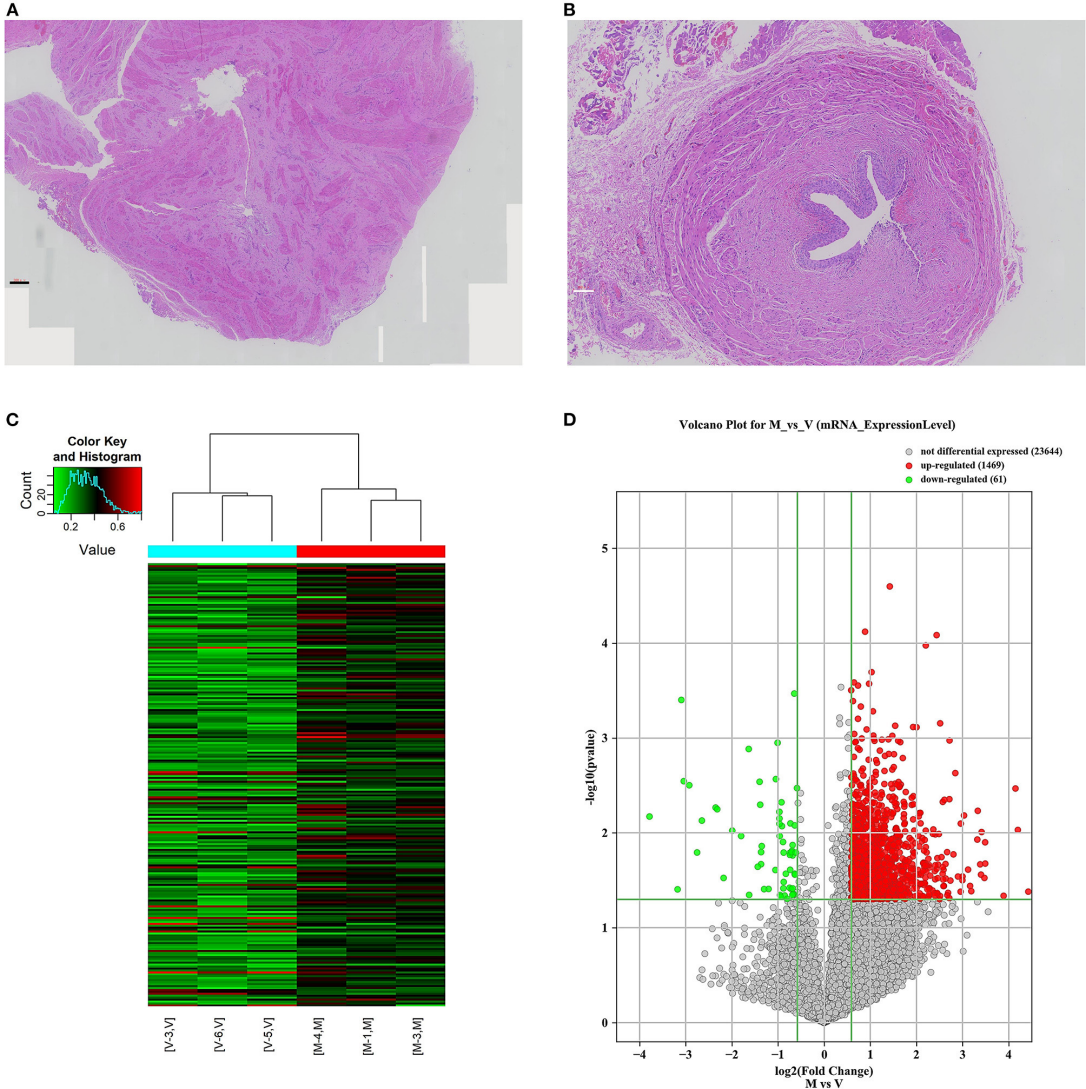
mRNA m^6^A modification profile changes and mRNA expression analysis in different ureteral phenotypes. **(A)** The ureter tissue from a patient with megaureter showed the atrophy and derangement in the muscular layers of specimen with increased extracellular matrix. **(B)** The ureter tissue from a patient with vesicoureteral reflux (VUR) showed no normal mucosal and muscular architecture. **(C)** Hierarchical clustering of all samples addressing the non-random partitioning of samples into two major groups: V-group (VUR), M-group (Megaureter). Each column represents one sample and each row represents one mRNA. **(D)** Volcano plot analysis of 1,469 upregulated and 61 downregulated mRNAs (M-group vs. V-group, *P* < 0.05). Red boxes represent ≥ 0.5-fold change difference, *P* < 0.05. Green boxes represent ≤ 0.7-fold change difference, *P* < 0.05.

A total of 24 ureteral samples were collected. The “V” group had 18 patients (male: female 11:7) with a median age of 42 months. There were seven cases of grade IV reflux, and 11 cases with grade V reflux. The “M” group had six patients (male: female 4:2) of megaureter with the median age of 47 months. Among the 24 cases, no pathogenic variants or copy number variants (CNVs) were identified through trio-whole exome sequencing. No marked differences were found in the morphology of the ureter tissue. The “M” group presented disruption in the muscular layers of specimens with an extracellular matrix accumulation. In the “V” group, there were no aberrant changes in the muscular layers and no rearrangement of collagen fibers, fibrocytes or fibroblasts in the adventitia (Figures 1A,B). Hence, we decided to explore the m^6^A patterns in the ureter tissue samples from patients with primary obstructive uropathy compared with those from the patients with VUR as controls.

### Distribution Patterns of m^6^A Methylation in Reflux or Obstructive Uropathy

**Figure 2:**
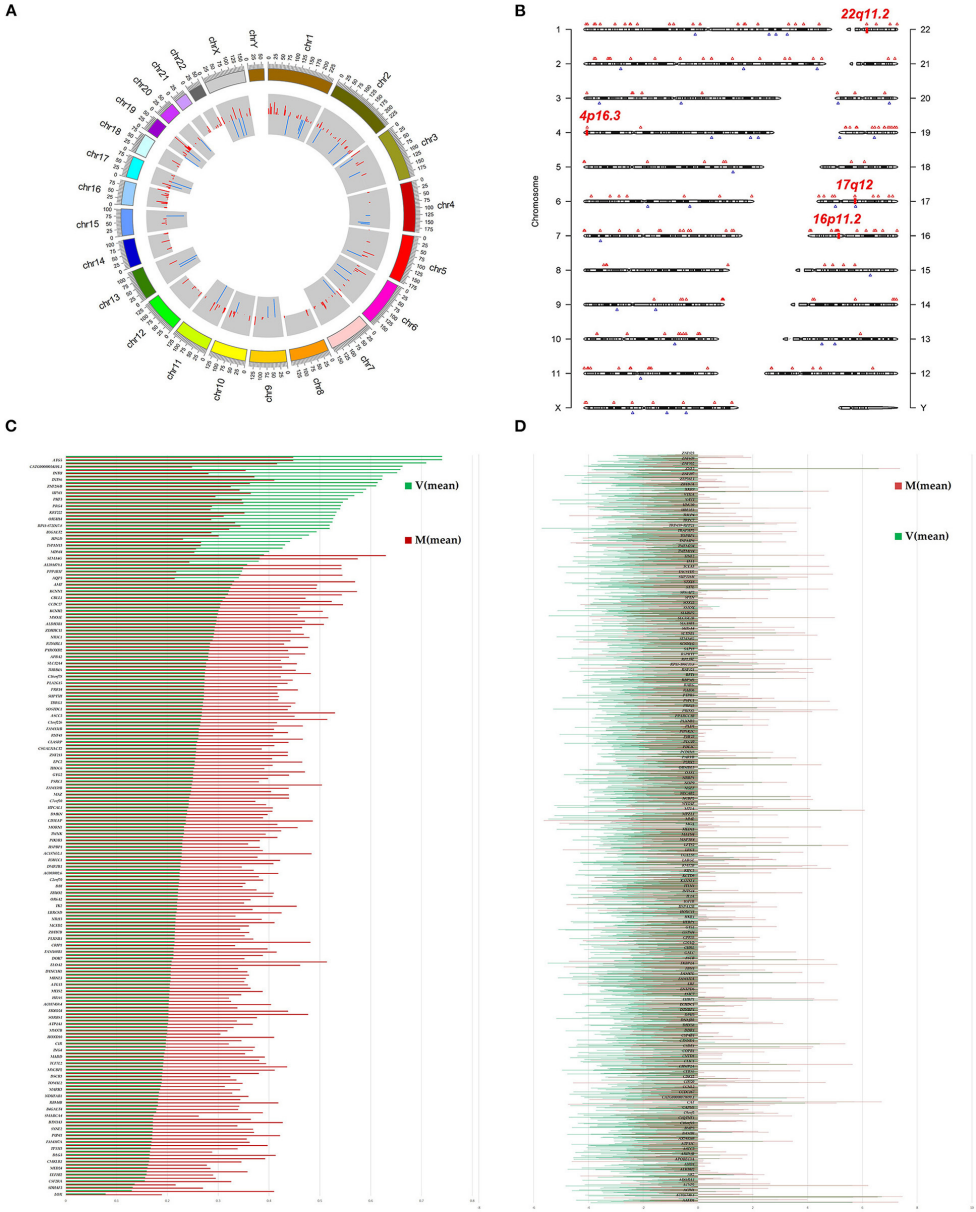
Landscape of *N6*-methyladenosine (m^6^A)-modified transcript in ureter tissue samples from patients with megaureter (M) or vesicoureteral reflux (VUR, V). **(A)** The m^6^A peaks' density distribution along the chromosomes. The first (red circle) and second (blue circle) tracks indicate the frequency distribution of the hyper-and hypomethylated peaks, respectively. chr, chromosome. **(B)** Chromosomal distribution patterns of the hypermethylated (red) and hypomethylated (blue) m6A peaks within mRNAs. **(C)** Differentially methylated mRNAs based on log2(FC). **(D)** Differentially expressed mRNAs based on log2(FC).

We investigated immunoprecipitated m^6^A methylation RNAs extracted from the ureters of patients with VUR and patients with obstructive megaureter disease to elucidate the regulation of transcript-specific m^6^A on ureteral phenotype. Microarray profiling revealed a difference in m^6^A methylation of 228 transcripts (228 mRNAs) between “V” (VUR) and “M” (Megaureter) groups (FC ≥ 1.5 or, ≤ 0.7) (Supplementary Tables S2, S3). The majority of the differentially methylated mRNAs (81.2%) were substantially hyper-methylated in the M/V comparison. The association between the samples were identified by hierarchical clustering, which were categorized based on m^6^A methylation level (Figures 1C,D). The peaks of the transcriptome-wide m^6^A in ureteral tissues were distributed across the 23 chromosomes shown in the Figure 2, indicating the broadly distributed peaks on the chromosomes. Additionally, the distribution pattern matched the density of gene content (Figures 2A,B). When the density of these differentially methylated peaks was calculated, it was found that they were not distributed homogeneously (chi square test, *P* < 0.05). Chromosomes 1, 2, 16, 19, 17, 7, and X were the top six chromosomes having the most methylated peaks. In detail, chromosome 1 (hypermethylated peaks, 22; hypomethylated peaks, 4) was followed by chromosome 2 (hypermethylated peaks, 18; hypomethylated peaks, 3), chromosome 16 (hypermethylated peaks, 19), chromosome 19 (hypermethylated peaks, 16; hypomethylated peaks, 2), chromosome 17 (hypermethylated peaks, 13; hypomethylated peaks, 2), chromosome 7 (hypermethylated peaks, 14; hypomethylated peaks, 1) and chromosome X (hypermethylated peaks, 10; hypomethylated peaks, 3). In the M/V comparison, most hypermethylated regions were enriched in chromosomes 1 (22 peaks), 2 (18 peaks), 16 (19 peaks), 19 (16 peaks) and 7 (14 peaks), whereas most hypomethylated peaks were enriched in chromosomes 1 (4 peaks), 4 (3 peaks), 2 (3 peaks), and X (3 peaks) (Figure 2A). Besides, the hypermethylated peaks with the maximal widths were located on chromosomes 14, 13, 7 and 5, whereas hypomethylated peaks with the maximal widths were observed on chromosomes 4, 6, 2 and 1. The peak site positions across the human chromosomes were displayed by mapping differentially methylated areas (inside mRNA transcripts) to the human chromosomes (Figure 2B). Ten hypermethylated peaks located in the *16p11.2* (6 hypermethylated peaks), *17q12* (2 hypermethylated peaks), *22q11.2* (1 hypermethylated peaks) and *4p16.3* (1 hypermethylated peaks), which are the known pathogenic CNVs for CAKUT. The top 20 differentially methylated mRNAs in M-group were listed in Table 1 based on log2 Fold change. The differently methylated genes located in the *17q12, 16p11.2, 22q11.2 and 4p16.3* were presented in the Supplementary Table S2, none of which matched the gene list associated with uropathy reported in the Enrichr metadata (20).

**Table 1:** The top 20 differentially methylated mRNAs based on log2(FC).

**Gene name**	**Chromosome**	**Peak start**	**Peak end**	***p*-value**	**Log_**2**_(FC)**	**Regulation**	**Methylated region**
*GYS1*	chr19	49471387	49496567	2.55E-03	3.16	Hyper	Exon
*DLG3*	chrX	69672155	69725337	3.47E-02	2.50	Hyper	Exon, Intron
*BTN3A3*	chr6	26440784	26452145	6.90E-03	2.49	Hyper	Intron
*PUM1*	chr1	31404353	31538551	5.38E-03	2.47	Hyper	Exon, Intron
*RPL13*	chr16	89627119	89630950	3.32E-02	2.47	Hyper	Exon
*LOX*	chr5	121398890	121413980	1.24E-02	2.41	Hyper	Exon
*HIRIP3*	chr16	30004311	30006964	2.72E-02	2.38	Hyper	Exon, Intron
*PEX14*	chr1	10534944	10690815	1.99E-02	2.36	Hyper	Exon, Intron
*IL5RA*	chr3	3133488	3151664	3.84E-02	2.36	Hyper	Exon, Intron
*RBM4B*	chr11	66432766	66445295	1.27E-03	2.31	Hyper	Exon, Intron
*RP11-872D17.8*	chr11	57154260	57158130	8.04E-03	0.66	hypo	Exon, Intron
*KRT222*	chr17	38811872	38821393	2.16E-02	0.66	hypo	Exon, Intron
*INTS6*	chr13	51939364	51995510	2.93E-02	0.66	hypo	Exon, Intron
*UGT1A6*	chr2	234601512	234681946	3.18E-02	0.64	hypo	Exon, Intron
*TMIGD3*	chr1	112026191	112106556	2.28E-02	0.64	hypo	Exon, Intron
*HDX*	chrX	83572886	83757461	2.58E-02	0.64	hypo	Exon, Intron
*MDM4*	chr1	204485507	204527247	4.76E-04	0.63	hypo	Exon, Intron
*MORF4L2*	chrX	102930428	102941746	5.72E-03	0.63	hypo	Exon
*AQP3*	chr9	33441806	33447551	3.44E-02	0.63	hypo	Exon
*PPP1R3F*	chrX	49126333	49143632	4.39E-02	0.63	hypo	Exon, Intron

### Differentially Methylated mRNAs in Reflux or Obstructive Uropathy

To explore the mRNA expression patterns between Megaureter and VUR, the total mRNAs tagged with Cy3 fluorescent dye were profiled using microarray. Most of the differentially methylated mRNAs were hypermethylated in the ureteral samples from M-group compared with that from V-group (196/228) (Figure 2C). The top 20 differentially methylated mRNAs with differential expressions were listed in Table 2.

**Table 2:** The top 20 differently methylated mRNAs with differential expressions.

**Gene name**	**Chromosome**	**Methylation regulation**			**Expression regulation**		
		**Log2(FC)**	***p*-value**	**Regulation**	**Log2(FC)**	***p*-value**	**Regulation**
*MAPK8IP3*	chr16	0.75	1.76E-02	Hyper	2.11	2.10E-03	Up
*MADD*	chr11	1.02	8.43E-03	Hyper	2.09	9.59E-03	Up
*RBM4B*	chr11	1.21	1.27E-03	Hyper	1.76	4.47E-02	Up
*OCEL1*	chr19	0.92	3.16E-03	Hyper	1.68	8.86E-03	Up
*DSP*	chr6	0.77	4.14E-02	Hyper	1.61	4.39E-02	Up
*MYCBP2*	chr13	1.11	8.65E-03	Hyper	1.56	2.53E-02	Up
*MORN1*	chr1	0.97	4.62E-02	Hyper	1.54	4.56E-02	Up
*SNRNP200*	chr2	0.76	2.03E-02	Hyper	1.49	4.63E-02	Up
*EWSR1*	chr22	0.76	4.65E-02	Hyper	1.47	1.83E-02	Up
*ZDHHC11*	chr5	0.60	2.64E-03	Hyper	1.45	3.21E-02	Up
*BTN3A3*	chr6	1.32	6.90E-03	Hyper	1.44	2.37E-02	Up
*TCF7L2*	chr10	1.04	4.93E-02	Hyper	1.37	2.80E-02	Down
*FAM131B*	chr7	0.65	9.98E-03	Hyper	1.30	4.28E-02	Up
*FAM160B1*	chr10	0.97	3.48E-02	Hyper	1.26	4.57E-02	Up
*TOM1L2*	chr17	1.15	4.12E-02	Hyper	1.26	1.71E-03	Up
*FXYD3*	chr19	−0.79	1.53E-02	Hypo	1.72	4.65E-02	Up
*SPOCK3*	chr4	−0.90	4.50E-02	Hypo	0.90	1.69E-02	Up
*TBX18*	chr6	−0.33	3.77E-02	Hypo	2.04	2.88E-02	Up
*BMP4*	chr14	−0.91	4.76E-02	Hypo	−0.88	3.84E-02	Down
*SOX2*	chr3	−0.99	3.40E-02	Hypo	−0.75	4.01E-02	Down

**Figure 3:**
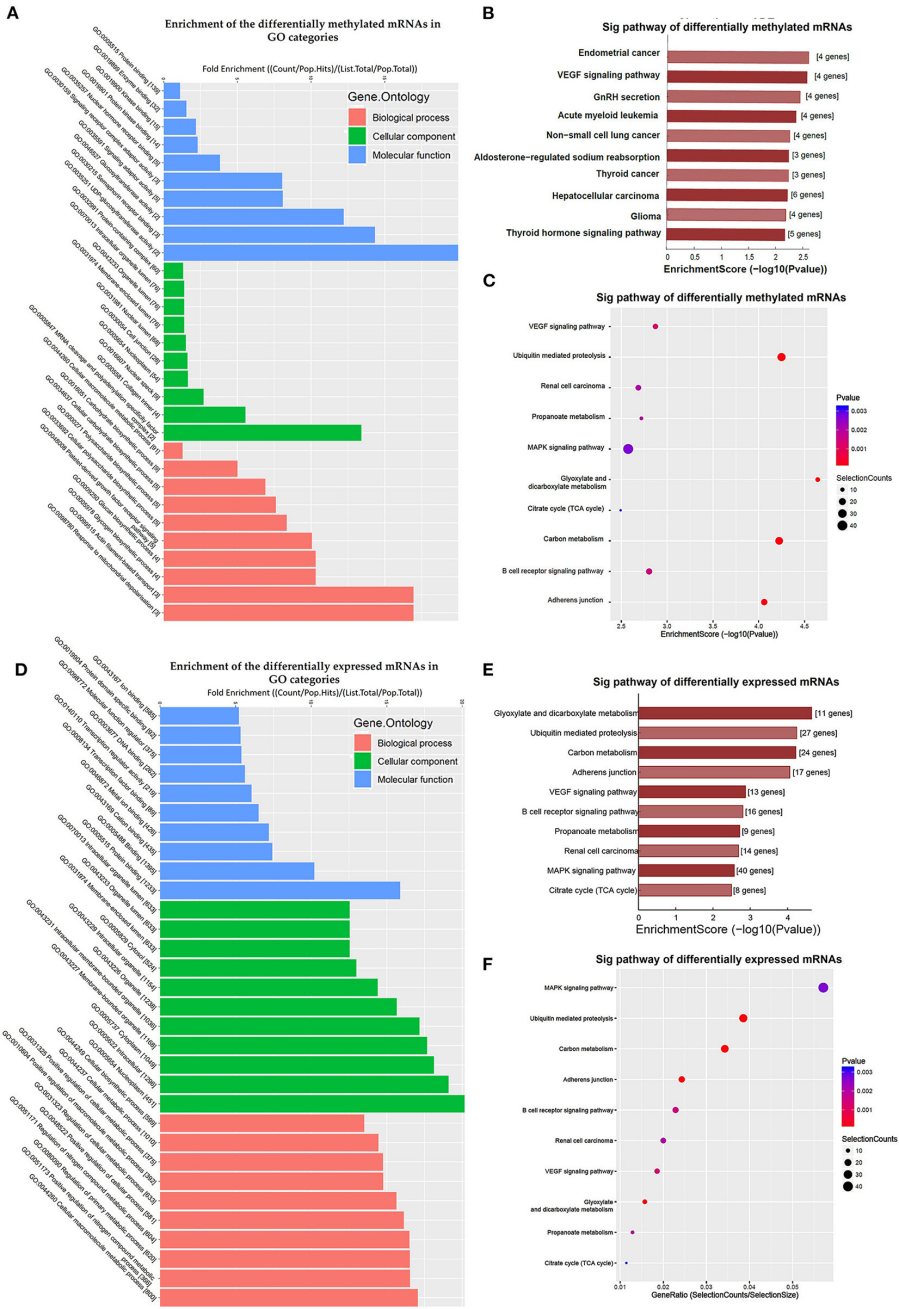
Clustering analyses of the differentially methylated mRNAs through Gene Ontology (GO) function and Kyoto Encyclopedia of Genes and Genomes (KEGG) pathway. **(A)** Enrichment of the differentially methylated mRNAs in GO categories such as biological process (BP), cellular component (CC), and molecular function (MF). **(B,C)** KEGG pathway involvement of differentially m^6^A mRNAs in patients with megaureter (M group) compared with vesicoureteral reflux (VUR, V group). **(D)** Enrichment of the differentially expressed mRNAs in GO categories such as BP, CC and MF. **(E,F)** KEGG pathway involvement of differentially expressed mRNAs in patients with the M group compared with the V group.

Additionally, GO and KEGG enrichment analyses were conducted to investigate the biological impact of the differentially methylated mRNAs on the uropathy pathogenic processes. A summary of the distributions of the differentially methylated mRNAs enriched in several GO categories was displayed in Figure 3A. In the biological process (BP) category, differentially methylated mRNAs were notably enriched in “actin filament-based transport” (fold enrichment 17.0, *P* = 0.7 × 10^−3^), “response to mitochondrial depolarization” (fold enrichment 17.0, *P* = 0.5 × 10^−3^), and “glycogen biosynthetic process” (fold enrichment 10.3, *P* = 0.7 × 10^−3^). In the cellular component (CC) category, the top three enriched functions were “mRNA cleavage and polyadenylation specificity factor complex” (fold enrichment 13.4, *P* = 0.010), “collagen trimer” (fold enrichment 5.5, *P* = 0.006), and “nuclear speck” (fold enrichment 2.7, *P* = 0.006). In the molecular function (MF) category, “UDP-glucosyltransferase activity” (fold enrichment 20.0, *P* = 0.004), “semaphorin receptor binding” and (fold enrichment 12.2, *P* = 0.011) were the most enriched terms. Likewise, the clustering analysis of the differentially methylated mRNAs were analyzed in the 10 KEGG categories (Figure 3B). The enrichment of KEGG pathways by the differentially methylated mRNAs included “Endometrial cancer”, “VEGF signaling pathway”, “GnRH secretion” and “Aldosterone-regulated sodium reabsorption” (*P* = 0.05, Figures 3B,C). Among these pathways, the *HRAS* and *PIK3R3* genes participated in most of the signaling pathways. And the *TCF7L2* gene as a key transcription factor in Wnt/β-catenins pathway participated in four of the signaling pathways shown by KEGG analysis. Further information on the enriched GO items, KEGG pathways and the corresponding genes were listed in Supplementary Tables S4,S5.

### Differentially Expressed Genes in Reflux or Obstructive Uropathy

Comparison of the expressed level of genes between the M group and the V group indicated 1530 differentially expressed genes in total (Supplementary Table S3), among which 1469 were upregulated and 61 were downregulated (Figure 2D). Among the differentially expressed genes, 116 genes matched the gene list associated with uropathy reported in the Enrichr platform (20) including *ACE, PAX2, RUNX2* (Supplementary Table S3). These differentially expressed genes were subsequently analyzed through GO and KEGG enrichment (Figures 3D–F). The findings of GO enrichment included “regulation of nitrogen compound metabolic process”, “regulation of macromolecule metabolic process”, “nucleoplasm”, “organelle lumen”, and “transcription factor binding” and “transcription regulator activity” (Figure 3D, Supplementary Table S6). KEGG analysis revealed several significantly enriched pathways involved in metabolism and development, such as “glyoxylate and dicarboxylate metabolism”, “Carbon metabolism”, “ubiquitin mediated proteolysis”, “MAPK signaling pathway”, “VEGF signaling pathway” and “adherens junction” (Figures 3E,F, Supplementary Table S7).

### Prediction of RNA-Binding Proteins

**Figure 4:**
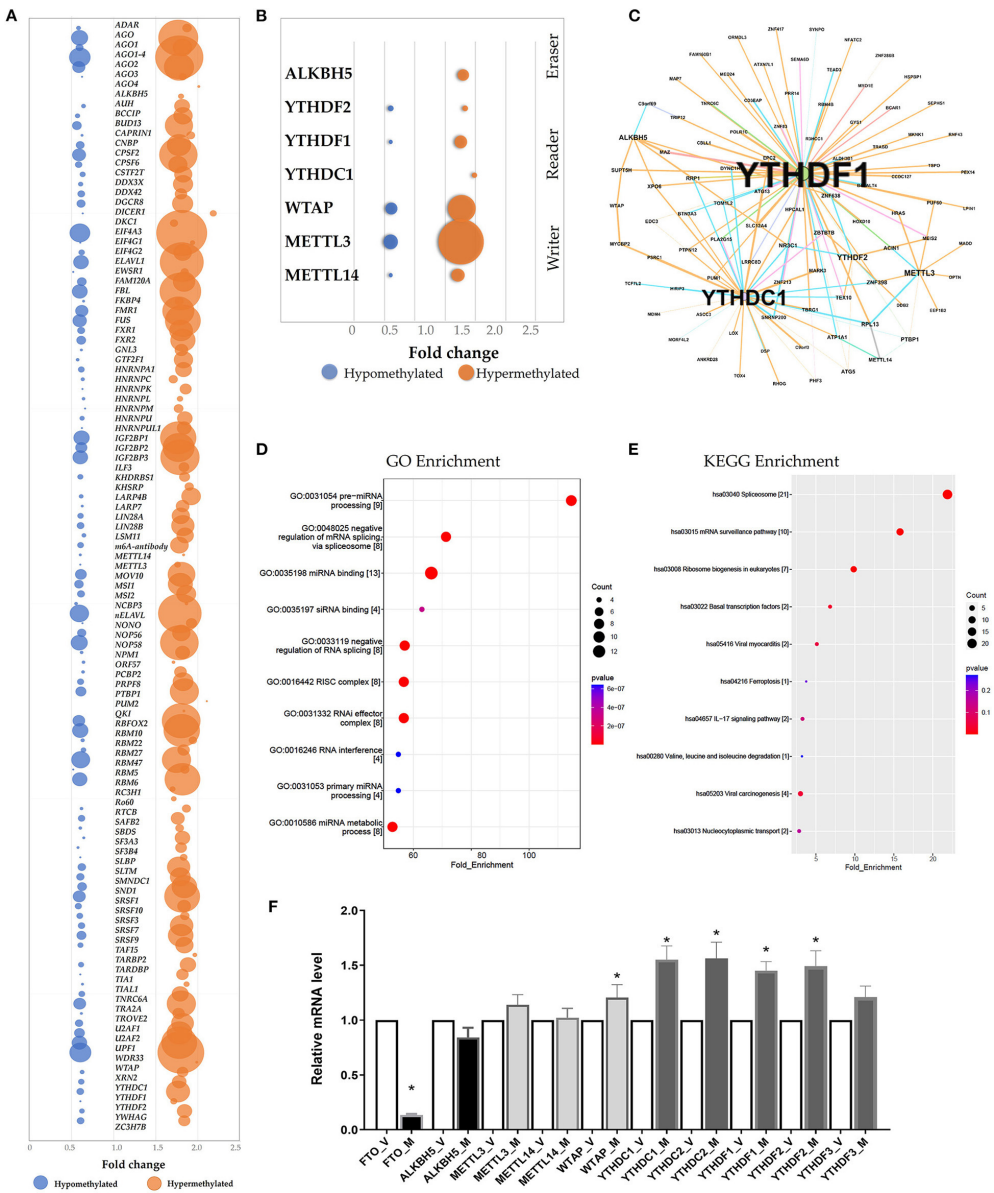
Prediction and function of RNA-binding proteins (RBPs) for *N*^6^-methyladenosine (m^6^A) methylation. **(A)** Bubble chart represented as the binding rates of the 140 RBPs. Values were displayed as the differentially methylated mRNAs based on log2(FC). The number of genes binding to the RBPs by prediction was presented by bubble size. **(B)** Bubble chart showing the binding rates of the known m^6^A readers (YTHDF1, YTHDF2, YTHDC1), m^6^A writers (WTAP, METTL3, METTL14) and eraser ALKBH5 by prediction. **(C)** Network mapping showing the genes binding to the m^6^A readers, writers and erasers by prediction. **(D,E)** Enrichment analyses of Gene Ontology (GO) and Kyoto Encyclopedia of Genes and Genomes (KEGG) of the 215 candidate RBP genes. **(F)** Real-time PCR confirmed the mRNA levels of m6A readers (YTHDC1, YTHDC2, YTHDF1, YTHDF2, YTHDF3), erasers (FTO and ALKBH5) and writers (WTAP, METTL3, METTL14).

The RMBase (v2.0) database was utilized to predict the RNA-Binding Proteins (RBPs) that might interact with the differentially methylated mRNAs (21). The hypermethylated regions yielded 113 candidate RBPs, whereas the hypomethylated regions yielded 102 candidate RBPs (Supplementary Table S8). We analyzed the RBPs binding abundance in the differentially methylated m^6^A regions. The RBPs that bound the most of regions were *EIF4A3, AGO, WDR33, ELAVL, IGF2BP1* and *FBL*. The RBPs in the hypermethylated group were mostly distributed in regions with log2(Fold change) 1.5-2.0, while RBPs in the hypomethylated group were mostly distributed in regions with log2 (Fold change) 0.5–1.0 (Figure 4A). RBP binding was more abundant in hypermethylated regions than in hypomethylated regions (Figure 4A), indicating that the RBPs might prefer to target hypermethylated gene sets. We found the known “Readers”, “Writers” and “Eraser” for m^6^A mostly distributed in the hyper-methylated regions included YTHDF1, YTHDF2, YTHDC1, METTL3, METTL14, WTAP and ALKBH5 (Figures 4B,C). Additionally, we also predated the enrichment of the RBPs associated with kidney disease including IGF2BP2 and MSI1 in the hypermethylated group.

According to GO and KEGG enrichment, these 215 RBPs were significantly enriched in GO items and KEGG pathways linked to the biogenesis and metabolism of RNA. For example, RBPs were significantly enriched in the GO items “Pre-miRNA processing”, “regulation of mRNA binding”, “RISC complex” and “RNAi effector complex” (Figure 4D, Supplementary Table S8). And the KEGG enrichment indicated the pathway in “spliceosome”, “mRNA surveillance pathway” and “Ribosome biogenesis” (Figure 4E, Supplementary Table S8).

Additionally, the mRNA levels of the m^6^A readers, writers and erasers were measured via qRT-PC. When the mRNA levels in the ureteral tissue samples from the M group were compared to that from the V group, the levels of YTHDF1, YTHDF2, YTHDC1, YTHDC2 and WTAP were significantly higher, whereas the level of FTO was significantly lower (*P* < 0.05; *n* = 6/group) (Figure 4F).

### Joint Profiling of m^6^A Methylation and Gene Expression in Reflux or Obstructive Uropathy

**Figure 5:**
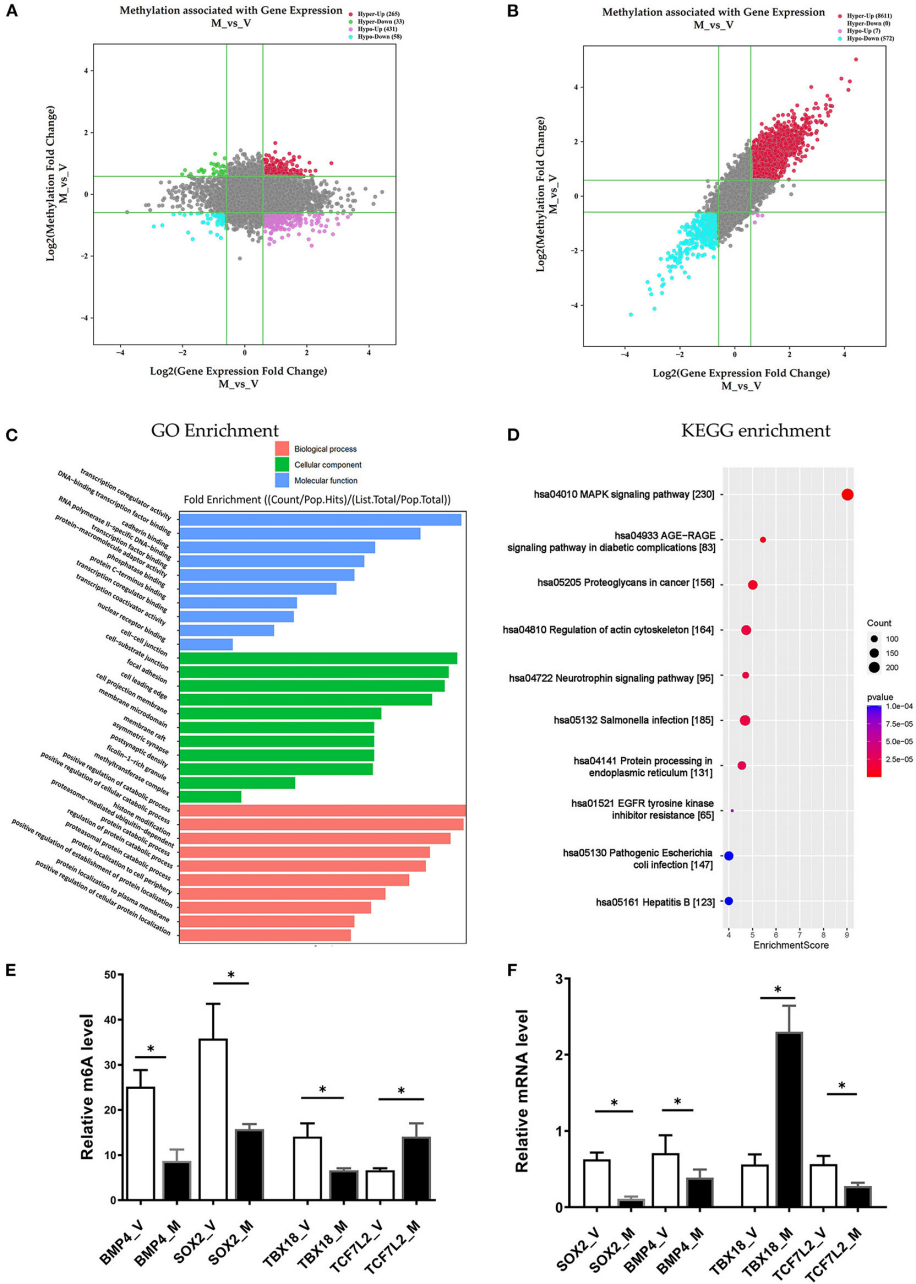
Conjoint analysis of m^6^A methylation and mRNA expression in different ureteral phenotypes. **(A)** Four-quadrant plot indicating the differentially methylated peaks within differentially expressed mRNAs (log2 FC> 0.5, *P* < 0.05). **(B)** Dot plot of Log2 fold change (FC) (mRNA expression) vs. Log2 FC (differential m^6^A methylation) revealing a positive association between total m^6^A methylation and level of mRNA expression (Pearson R^2^ = 0.20, *P* < 0.05). **(C,D)** Enrichment analyses of mRNAs with both differential methylation and expression via Gene Ontology (GO) and Kyoto Encyclopedia of Genes and Genomes (KEGG). **(E)** MeRIP-qPCR validation of m^6^A level changes in four hyper-methylated or hypo-methylated genes in megaureter group (M) and vesicoureteral reflux (VUR) group (V) samples. **(F)** Relative mRNA levels of four genes were assessed by real-time PCR in megaureter group (M) and vesicoureteral reflux (VUR) group (V) samples. Data are expressed as mean ± SD; data were analyzed using the nonparametric *t*-test. *P < 0.05; M group vs. V group (*n* = 6 per group). Hypo, hypomethylation; Hyper, hypermethylation; Up, upregulated expression; Down, downregulated expression.

In the M/V comparison, joint analysis of the differentially methylated m^6^A mRNAs and differentially expressed mRNA revealed the four modes of m^6^A modification-associated mRNAs: (i) m^6^A hyper-methylated and upregulated mRNAs; (ii) m^6^A hyper-methylated and downregulated mRNAs; (iii) m^6^A hypo-methylated and upregulated mRNAs, (iv) m^6^A hypo-methylated and downregulated mRNAs. We teased out the significant differential expressions in 787 differentially methylated mRNA transcripts. Among the 298 hypermethylated mRNAs, we found 265 upregulated and 33 downregulated expressed mRNAs. Among the 489 hypomethylated mRNAs, we found 431 upregulated and 58 downregulated expressed mRNAs (Figure 5A, Supplementary Table S9). The methylation levels of these 787 mRNAs were positively correlated with their expression levels according to Pearson's correlation analysis (R^2^ = 0.20, *P* < 0.05) (Figure 5B). It indicated the crucial role of m^6^A methylation in the gene expression regulation in uropathy.

Likewise, we performed GO and KEGG enrichment analyses (Figures 5C,D, Supplementary Table S10). GO analysis showed that “cell-substrate junction”, “focal adhesion” and “cell leading edge” are major cellular component; “protein localization to cell periphery”, “proteasome-mediated ubiquitin-dependent protein catabolic process” and “positive regulation of catabolic process” are major biological processes; “DNA-binding transcription factor binding”, “RNA polymerase II-specific DNA-binding transcription factor binding” and “transcription coregulator activity” are major molecular functions of differentially m^6^A methylated transcripts. KEGG analysis also showed that “MAPK signaling”, “AGE–RAGE signaling pathway”, “EGFR tyrosine kinase inhibitor resistance”, and “regulation of actin cytoskeleton” are the major pathways associated with the differentially m^6^A methylated transcripts in megaureter obstructive uropathy.

### Validation of the Microarray Data by MeRIP and qRT-PCR Analyses

To confirm the m^6^A mRNA microarray findings in ureteral tissues, we employed MeRIP to detect the m^6^A levels of four mRNAs involved in the pathway regulatory network, which matched the gene list associated with uropathy reported in the Enrichr metadata (20). As shown in Figure 5, the m^6^A levels of BMP4, TBX18 and SOX2 mRNAs were lower in the ureter samples from patients with megaureter (M group) than those from patients with VUR (V group). While the m^6^A levels of TCF7L2 mRNAs in the M group were higher than those in the V group (Figure 5E). Next, we detected the expression levels via qRT-PCR to demonstrate whether mRNA expression was correlated with m^6^A modification. It revealed that the mRNAs of BMP4, SOX2 and TCF7L2 were downregulated in the M group, but the mRNAs of TBX18 were upregulated in the M group (Figure 5F). The results indicated that hypo-methylated TBX18 may induce the stability of mRNA expression, while hyper-methylated TCF7L2 may display a low level of mRNA expression. The m^6^A modification was also associated with the BMP4 and SOX2 mRNAs levels (Figures 5E,F).

## Discussion

Despite the growing knowledge of the impact of the m^6^A modifications on mRNA degradation and translation, it is still unclear how the modifications affect organ development. We provided revealing insights into the m^6^A modification patterns in ureter tissue samples from patients with obstructive megaureter and patients with VUR. The analysis of the uropathy-related methylated genes and their potential functions enlighten us the unique pathogenesis of obstructive and reflux uropathy.

Advances in clinical diagnostics and molecular techniques have helped us better understand several causes of CAKUT. Increasing evidence suggests that kidney and urinary tract development is controlled by classical signaling pathways, as well as by epigenetic mechanisms involving chromatin histone modifications, DNA methylation and RNA modification (18). m^6^A has a broad range of functions in organ development and disease processing (22, 23). Several studies have delineated the regulation mechanism of m^6^A modification in glomerulopathy and renal fibrosis (16, 24). Thus far, few studies have investigated the involvement of m^6^A in kidney and ureter development. Hydronephrosis is the most common features in CAKUT which can be caused by obstructive uropathy or reflux uropathy. A cause of obstructive uropathy in children is primary megaureter, which represents a ureter with a larger diameter than usual, resulting in vesicoureteral junction obstruction. The morpho-pathogenesis of megaureter is characterized by apoptotic epitheliums and smooth muscle cells, as well as tissue deterioration in the ureter epithelium and connective tissue (25). Primary VUR represents the retrograde flow of urine from the bladder into the ureter, resulting in reflux nephropathy. Refluxing ureters are distinguished by lesions at the vesico-ureteric junction, which includes disordered smooth muscle fibers and the destruction of smooth muscle cell structure, resulting in valve incompetence. (26). The mechanism of primary megaureter and VUR is poorly understood and remains controversial. Here, the m^6^A methylomic landscape of ureters was studied to get insight into the megaureter and VUR.

In this study, MeRIP combined with microarray analysis revealed the different m^6^A methylation patterns in ureters between the megaureter and VUR. The majority of the differentially methylated mRNAs in the megaureter (M) group were substantially hyper-methylated compared to the VUR (V) group. Mapping the differentially methylated regions with visualized the peak site locations in the chromosomes showed the enrichment of methylation in chromosomes 1, 2, 16, 19, 17, 7, and X. There were 10 methylated peaks located in the *17q12, 16p11.2, 22q11.2* and *4p16.3* which are the known pathogenic CNVs for CAKUT. Pathogenic CNVs have been identified in 22.5% of patients with syndromic deformities and in 14.5% of patients with isolated CAKUT (27). It has been documented that large deletions were mostly detected in upper urinary tract disorders (e.g., kidney agenesis or dysplasia), whereas duplications were more common in lower urinary tract phenotypes (e.g., duplex kidney, VUR, or posterior urethral valves) (18). Although the known pathogenic CNVs were not detected in the participants in our study, the enrichment of m^6^A modifications in these regions may participate in the development of the ureteric bud (UB) as the origin of lower urinary tract. Further exploration on the genes and long noncoding RNA (lncRNA) located the CNVs regions with multiple methylated peaks may provide more information of pathogenesis of uropathy.

A total of 228 mRNAs were revealed to be substantially differential methylated in the M/V comparison. Understanding the specific m^6^A-modified transcripts provided the clues to delineate the pathogenesis between obstructive uropathy and reflux uropathy. GO and KEGG pathway analysis indicated several pathways. Among them, the *HRAS, PIK3R3* and *TCF7L2* genes participated in most of the signaling pathways. A case with PUV has identified the pathogenic CNVs (deletion at *1p34.1*), which covers both *PIK3R3* and *TSPAN1* genes' exonic regions (28). PIK3R3 represents a regulatory subunit of PI3K which coordinates multiple cell functions such as cell migration, cell proliferation, and cell survival. Members of the Ras superfamily can also modulate cell growth, differentiation, proliferation, and migration. It was demonstrated that UB cells with R-Ras–expressing participated in cell growth and branching morphology. H-Ras–expressing UB cells had a high ability to migrate forming long unbranched tubules, whereas TC21-expressing UB cells were characterized with branching excessively with a reduced capacity to migrate (29). Additionally, the diabetes gene and Wnt pathway effector *TCF7L2* may increase susceptibility to both diabetes and kidney disease (30). Hence, the differentially methylated mRNAs provide the clues for understanding the distinct pathogenesis of uropathy.

Joint analysis of the methylated mRNAs with expression level revealed a large proportion of hypermethylated mRNAs with high expression in the ureters of patients with uropathy. It indicated the role of m^6^A methylation on the of mRNAs stability. However, some differential mRNAs in the M/V comparison, such as hypermethylated mRNAs with downregulated expression may contribute to uropathy as well, despite accounting for a minor proportion of the total. Notably, we confirmed the downregulated expression of *TCF7L2* as hypermethylated mRNAs, the upregulated expression of *TBX18* as hypomethylated mRNAs, the downregulated expression of *BMP4* and *SOX2* as hypomethylated mRNAs in the M group compared with the V group. We selected these four representative mRNAs (*TCF7L2, TBX18, BMP4* and *SOX2*) from the key pathways associated with the differentially m^6^A-methylated mRNAs in uropathy. *TCF7L1/TCF7L2* complexes were confirmed as a β-catenin-driven switcher to enhance the differentiation-promoting target genes during the initiation of nephron progenitor cells (31). *Tbx18* is expressed in the mesenchymal compartment of the ureter of the fetal mouse (32), and mutations of *TBX18* have been identified as the pathogenic variants of CAKUT (33). *BMP4*, a member of the TGF-β superfamily, plays a key role in the early phase of the kidney and urinary tract morphogenesis. It can restrain ectopic budding from the UB or the ureter stalk by blocking inductive signals from the metanephric mesenchyme. It may also contribute to the branching process of metanephros' ureter (34). *SOX2* is a key transcription factor during the organ development that can control the pluripotency of early embryonic cells. SOX2 anophthalmia syndrome is an autosomal dominant disorder characterized by severe developmental eye deformities as well as abnormalities in esophageal, brain, genital, and kidney abnormalities (35).

Cellular m^6^A homeostasis is maintained through coordinating the activity of m^6^A methylase complex (i.e., writers), demethylases (i.e., erasers) and the m^6^A binding proteins (i.e., readers) to regulate RNA fate. Currently, the differential m^6^A methylation in uropathy was associated with the decline of the subunits of the m^6^A erasers (FTO) and the increase of the readers (YTHDF1, YTHDF2, YTHDC1 and YTHDC2). Interestingly, FTO showed the highest change of mRNA level between the M group and V group of all the m^6^A regulators tested. It has been reported loss of FTO antagonized Wnt signaling resulting in development defeats in mice (36). FTO can also modulated fibrogenic response in mouse models of obstructive nephropathy (37). We cannot fully explain the enhancement of all the m^6^A regulators. Further detecting the m^6^A modification of space-time could gain more information on epigenetic modulation during different phase of UB development and disease processing.

RNA-binding proteins (RBPs) are of crucial role in post-transcriptional gene regulation and protein synthesis. RBPs contribute to a wide spectrum of kidney disease, including glomerular disease, diabetic kidney disease and cystic kidney disease. RNA interactome capture (RIC) can identify RBPs bound to polyA-tailed transcripts (38). RBPs recognize m^6^A sites with a high degree of specificity (39). We predicted the candidate RBPs binding to the methylated peak sites using the RMBase database (21). Subsequent results showed that the binding abundance of RBPs in the hypermethylated regions. This hinted that the RBPs prefer to target hypermethylated peaks. Concordantly, the m^6^A regulators (YTHDF1, YTHDF2, YTHDC1, METTL3, METTL14, WTAP and ALKBH5) were mostly distributed in the hyper-methylated regions. The prediction of the enrichment of the RBPs associated with kidney disorders (40) was laid out with *IGF2BP2* and *MSI1* in the hypermethylated group. Downregulation o*f MSI1* can induce tubulointerstitial fibrosis (41). *IGF2BP2* was involved in the damage of glomerular basement membrane (42). We speculated that RBPs may regulate RNA processes and consequently play a role in the pathogenesis of uropathy by controlling the differentially methylated transcripts.

This study had potential limitations. First, we could not examine the expression level of all the m^6^A-related genes and RBPs in the ureter tissue because of the limitation of specimens from pediatric patients. We should start more work on the possible crosstalk between the differentially methylated genes and RBPs, as well as the mechanism of RBPs regulating the gene expression. Functional studies of the candidate genes in various animal models of uropathy need to be conducted to examine the different expression levels during the development course. Second, although the target genes modified by m^6^A were outlined, the process of methylation readers, erasers or writers regulating the target genes was not delineated. Further studies should be conducted to investigate whether readers, erasers or writers regulate the stability, translation efficiency, or degradation of target genes. In addition, the involvement of the differentially methylated mRNAs and the m^6^A-related enzymes and RBPs in the other types of CAKUT such as renal dysplasia and cystic renal disease deserve further investigation. The common changes in methylated mRNAs in both obstructive nephropathy and reflux nephropathy are to be detected in the renal parenchymal compared with normal control, which may provide novel findings on the epigenetic mechanisms on renal development.

In conclusion, we presented a summary of differentially methylated mRNAs and their potential binding proteins, which might be key regulators in the development of obstructive and reflux uropathy. To our knowledge, we provided the initial m^6^A methylomic landscape in the ureter tissue of children, which highlighted a new direction for unraveling the distinct mechanism of m^6^A methylation in uropathy.

## Data Availability Statement

The data discussed in this publication have been deposited in NCBI's Gene Expression Omnibus and are accessible through GEO Series accession number GSE195849 (https://www.ncbi.nlm.nih.gov/geo/query/acc.cgi?acc=GSE195849).

## Ethics Statement

The studies involving human participants were reviewed and approved by No. 2020_363. Written informed consent to participate in this study was provided by the participants' legal guardian/next of kin.

## Author Contributions

JR and HX led and supervised the project and were involved in all aspects of the study. JR, XW, and HS conceived and designed the experiments. HS, TX, JF, XY, YL, YF, LX, QQ, JS, QS, and LT performed the experiments. JR and TX analyzed the data, interpreted the results, and prepared the figures. HS and JR wrote the paper with input from QQ. All authors commented and made edits to the manuscript and contributed to the article and approved the final version.

## Funding

JR is supported by a grant from the National Key Research and Development Program of China (2021YFC2701100), a grant from Clinical Research Plan of SHDC(SHDC2020CR2064B), and a grant from National Natural Science Foundation of China (NSFC-8182207). HX is supported by a grant from National Natural Science Foundation of China (NSFC-81873593).

## Conflict of Interest

The authors declare that the research was conducted in the absence of any commercial or financial relationships that could be construed as a potential conflict of interest.

## Publisher's Note

All claims expressed in this article are solely those of the authors and do not necessarily represent those of their affiliated organizations, or those of the publisher, the editors and the reviewers. Any product that may be evaluated in this article, or claim that may be made by its manufacturer, is not guaranteed or endorsed by the publisher.

## References

[1] Wang X Zhao BS Roundtree IA Lu Z Han D Ma H. N(6)-methyladenosine modulates messenger RNA translation efficiency. Cell. (2015) 161:1388–99. 10.1016/j.cell.2015.05.014 26046440 PMC4825696

[2] Liu J Yue Y Han D Wang X Fu Y Zhang L. A METTL3-METTL14 complex mediates mammalian nuclear RNA N6-adenosine methylation. Nat Chem Biol. (2014) 10:93–5. 10.1038/nchembio.1432 24316715 PMC3911877

[3] Ping X-L Sun B-F Wang L Xiao W Yang X Wang W-J. Mammalian WTAP is a regulatory subunit of the RNA N6-methyladenosine methyltransferase. Cell Res. (2014) 24:177–89. 10.1038/cr.2014.3 24407421 PMC3915904

[4] Jia G Fu Y Zhao X Dai Q Zheng G Yang Y. N6-methyladenosine in nuclear RNA is a major substrate of the obesity-associated FTO. Nat Chem Biol. (2011) 7:885–7. 10.1038/nchembio.687 22002720 PMC3218240

[5] Zheng G Dahl JA Niu Y Fedorcsak P Huang C-M Li CJ. ALKBH5 is a mammalian RNA demethylase that impacts RNA metabolism and mouse fertility. Mol Cell. (2013) 49:18–29. 10.1016/j.molcel.2012.10.015 23177736 PMC3646334

[6] Wang X Lu Z Gomez A Hon GC Yue Y Han D. N6-methyladenosine-dependent regulation of messenger RNA stability. Nature. (2014) 505:117–20. 10.1038/nature12730 24284625 PMC3877715

[7] Frye M Harada BT Behm M He C. RNA Modifications modulate gene expression during development. Science. (2018) 361:1346–9. 10.1126/science.aau1646 30262497 PMC6436390

[8] Zhao L-Y Song J Liu Y Song C-X Yi C. Mapping the epigenetic modifications of DNA and RNA. Protein Cell. (2020) 11:792–808. 10.1007/s13238-020-00733-7 32440736 PMC7647981

[9] Mathiyalagan P Adamiak M Mayourian J Sassi Y Liang Y Agarwal N. FTO-Dependent N6-methyladenosine regulates cardiac function during remodeling and repair. Circulation. (2019) 139:518–32. 10.1161/CIRCULATIONAHA.118.033794 29997116 PMC6400591

[10] Gu HF. Genetic and epigenetic studies in diabetic kidney disease. Front Genet. (2019) 10:507. 10.3389/fgene.2019.00507 31231424 PMC6566106

[11] Wang J-N Wang F Ke J Li Z Xu C-H Yang Q. Inhibition of METTL3 attenuates renal injury and inflammation by alleviating TAB3 m6A modifications via IGF2BP2-dependent mechanisms. Sci Transl Med. (2022) 14:eabk2709. 10.1126/scitranslmed.abk2709 35417191

[12] Teng F Tang W Wuniqiemu T Qin J Zhou Y Huang X. N6-Methyladenosine methylomic landscape of lung tissues in murine acute allergic asthma. Front Immunol. (2021) 12:740571. 10.3389/fimmu.2021.740571 34737744 PMC8560743

[13] Martini S Eichinger F Nair V Kretzler M. Defining human diabetic nephropathy on the molecular level: integration of transcriptomic profiles with biological knowledge. Rev Endocr Metab Disord. (2008) 9:267–74. 10.1007/s11154-008-9103-3 18704688 PMC2597685

[14] Lu Z Liu H Song N Liang Y Zhu J Chen J. METTL14 aggravates podocyte injury and glomerulopathy progression through N6-methyladenosine-dependent downregulating of Sirt1. Cell Death Dis. (2021) 12:881. 10.1038/s41419-021-04156-y 34580283 PMC8476597

[15] Xu Y Yuan XD Wu JJ Chen RY Xia L Zhang M. The N6-methyladenosine mRNA methylase METTL14 promotes renal ischemic reperfusion injury via suppressing YAP1. J Cell Biochem. (2020) 121:524–33. 10.1002/jcb.29258 31318098

[16] Xu Z Jia K Wang H Gao F Zhao S Li F. METTL14-regulated PI3K/Akt signaling pathway via PTEN affects HDAC5-mediated epithelial-mesenchymal transition of renal tubular cells in diabetic kidney disease. Cell Death Dis. (2021) 12:32. 10.1038/s41419-020-03312-0 33414476 PMC7791055

[17] Murugapoopathy V Gupta IR. A primer on congenital anomalies of the kidneys and urinary tracts (CAKUT). Clin J Am Soc Nephrol. (2020) 15:723–31. 10.2215/CJN.12581019 32188635 PMC7269211

[18] Khan K Ahram DF Liu YP Westland R Sampogna RV Katsanis N. Multidisciplinary approaches for elucidating genetics and molecular pathogenesis of urinary tract malformations. Kidney Int. (2021). 10.1016/j.kint.2021.09.034 34780871 PMC8934530

[19] International Reflux Study Committee. Medical versus surgical treatment of primary vesicoureteral reflux. Pediatrics. (1981) 67:392–400. 10.1542/peds.67.3.392 7017578

[20] Chen EY Tan CM Kou Y Duan Q Wang Z Vaz Meirelles G. Enrichr: interactive and collaborative HTML5 gene list enrichment analysis tool: BMC Bioinformatics. BMC Bioinformatics. (2013) 14:128. 10.1186/1471-2105-14-128 23586463 PMC3637064

[21] Sun W-J Li J-H Liu S Wu J Zhou H Qu L-H. RMBase: a resource for decoding the landscape of RNA modifications from high-throughput sequencing data. Nucleic Acids Res. (2016) 44:D259–65. 10.1093/nar/gkv1036 26464443 PMC4702777

[22] Xiao S Cao S Huang Q Xia L Deng M Yang M. The RNA N6-methyladenosine modification landscape of human fetal tissues. Nat Cell Biol. (2019) 21:651–61. 10.1038/s41556-019-0315-4 31036937

[23] Tang Y Chen K Song B Ma J Wu X Xu Q. m6A-Atlas: a comprehensive knowledgebase for unraveling the N6-methyladenosine (m6A) epitranscriptome. Nucleic Acids Res. (2021) 49:D134–43. 10.1093/nar/gkaa692 32821938 PMC7779050

[24] Zhao H Pan S Duan J Liu F Li G Liu D. Integrative analysis of m6A regulator-mediated RNA methylation modification patterns and immune characteristics in lupus nephritis. Front Cell Dev Biol. (2021) 9:724837. 10.3389/fcell.2021.724837 34557492 PMC8454410

[25] Walker KA Sims-Lucas S Bates CM. Fibroblast growth factor receptor signaling in kidney and lower urinary tract development. Pediatr Nephrol. (2016) 31:885–95. 10.1007/s00467-015-3151-1 26293980 PMC4761523

[26] Sofikerim M Sargon M Oruc O Dogan HS Tekgul S. An electron microscopic examination of the intravesical ureter in children with primary vesico-ureteric reflux. BJU Int. (2007) 99:1127–31. 10.1111/j.1464-410X.2007.06751.x 17309556

[27] Verbitsky M Westland R Perez A Kiryluk K Liu Q Krithivasan P. The copy number variation landscape of congenital anomalies of the kidney and urinary tract. Nat Genet. (2019) 51:117–27. 10.1038/s41588-018-0281-y 30578417 PMC6668343

[28] Boghossian NS Sicko RJ Kay DM Rigler SL Caggana M Tsai MY. Rare copy number variants implicated in posterior urethral valves. Am J Med Genet A. (2016) 170:622–33. 10.1002/ajmg.a.37493 26663319 PMC6205289

[29] Pozzi A Coffa S Bulus N Zhu W Chen D Chen X. R-Ras, and TC21 differentially regulate ureteric bud cell branching morphogenesis. Mol Biol Cell. (2006) 17:2046–56. 10.1091/mbc.e05-08-0800 16467383 PMC1415315

[30] Del Bosque-Plata L Martínez-Martínez E Espinoza-Camacho MÁ Gragnoli C. The role of TCF7L2 in type 2 diabetes. Diabetes. (2021) 70:1220–8. 10.2337/db20-0573 34016596 PMC8275893

[31] Guo Q Kim A Li B Ransick A Bugacov H Chen X. A β-catenin-driven switch in TCF/LEF transcription factor binding to DNA target sites promotes commitment of mammalian nephron progenitor cells. Elife. (2021) 10. 10.7554/eLife.64444.sa2 33587034 PMC7924951

[32] Airik R Bussen M Singh MK Petry M Kispert A. Tbx18 regulates the development of the ureteral mesenchyme. J Clin Invest. (2006) 116:663–74. 10.1172/JCI26027 16511601 PMC1386107

[33] Vivante A Kleppa M-J Schulz J Kohl S Sharma A Chen J. Mutations in TBX18 cause dominant urinary tract malformations via transcriptional dysregulation of ureter development. Am J Hum Genet. (2015) 97:291–301. 10.1016/j.ajhg.2015.07.001 26235987 PMC4862256

[34] Y Miyazaki K Oshima A Fogo B L Hogan I Ichikawa. Bone morphogenetic protein 4 regulates the budding site and elongation of the mouse ureter. J Clin Invest. 105:863–73. 10.1172/JCI8256 10749566 PMC377476

[35] Bakrania P Robinson DO Bunyan DJ Salt A Martin A Crolla JA. SOX2 anophthalmia syndrome: 12 new cases demonstrating broader phenotype and high frequency of large gene deletions. Br J Ophthalmol. (2007) 91:1471–6. 10.1136/bjo.2007.117929 17522144 PMC2095460

[36] Osborn DP Roccasecca RM McMurray F Hernandez-Hernandez V Mukherjee S Barroso I. Loss of FTO antagonises Wnt signaling and leads to developmental defects associated with ciliopathies. PLoS ONE. (2014) 9:e87662. 10.1371/journal.pone.0087662 24503721 PMC3913654

[37] Wang C-Y Shie S-S Tsai M-L Yang C-H Hung K-C Wang C-C. FTO modulates fibrogenic responses in obstructive nephropathy. Sci Rep. (2016) 6:18874. 10.1038/srep18874 26727661 PMC4698750

[38] Castello A Fischer B Eichelbaum K Horos R Beckmann BM Strein C. Insights into RNA biology from an atlas of mammalian mRNA-binding proteins. Cell. (2012) 149:1393–406. 10.1016/j.cell.2012.04.031 22658674

[39] Zhang Z Luo K Zou Z Qiu M Tian J Sieh L. Genetic analyses support the contribution of mRNA N6-methyladenosine (m6A) modification to human disease heritability. Nat Genet. (2020) 52:939–49. 10.1038/s41588-020-0644-z 32601472 PMC7483307

[40] Seufert L Benzing T Ignarski M Müller R-U. RNA-binding proteins and their role in kidney disease. Nat Rev Nephrol. (2021). 10.1038/s41581-021-00497-1 34732838

[41] Jadhav S Ajay AK Trivedi P Seematti J Pellegrini K Craciun F. RNA-binding protein Musashi homologue 1 regulates kidney fibrosis by translational inhibition of p21 and Numb mRNA. J Biol Chem. (2016) 291:14085–94. 10.1074/jbc.M115.713289 27129280 PMC4933168

[42] Schaeffer V Hansen KM Morris DR LeBoeuf RC Abrass CK. RNA-binding protein IGF2BP2/IMP2 is required for laminin-β2 mRNA translation and is modulated by glucose concentration. Am J Physiol Renal Physiol. (2012) 303:F75–82. 10.1152/ajprenal.00185.2012 22513850 PMC3431147

